# Aerodynamic barriers to inhaled furosemide delivery during dyspnoea revealed by experimental and computational modelling

**DOI:** 10.1038/s41598-026-55439-3

**Published:** 2026-06-08

**Authors:** Frantisek Lizal, Miloslav Belka, Zdenka Sklubalova, Ondrej Misik, Jakub Elcner, Michal Svarc, Ludmila Matysova, Vladimir Koblizek

**Affiliations:** 1 Faculty of Mechanical Engineering, Energy Institute, Brno University of Technology, Technicka 2896/2, 616 69 Brno, Czech Republic; 2 Faculty of Pharmacy, Department of Analytical Chemistry, Charles University, Hradec Kralove, Czech Republic; 3 Faculty of Pharmacy, Department of Pharmaceutical Technology, Charles University, Hradec Kralove, Czech Republic; 4 Department of Pulmonology, University Hospital Hradec Kralove, Hradec Kralove, Czech Republic

**Keywords:** Dyspnoea, Inhaled furosemide, Vibrating mesh nebuliser, Airway model, Computational fluid dynamics, Drug delivery, Diseases, Engineering, Medical research

## Abstract

Inhaled furosemide shows potential for relieving refractory dyspnoea, yet clinical trials have yielded inconsistent results. We hypothesise this failure stems from a “Dyspnoea Paradox”, where high inspiratory flows create an aerodynamic barrier. We evaluated furosemide delivery using a vibrating mesh nebuliser under simulated dyspnoeic conditions (tidal volume 1.39 L, frequency 28.6 min⁻¹). The study integrated standard Pharmacopoeial characterisation, in vitro deposition in a realistic human airway replica, and computational modelling (Large Eddy Simulation and Multiple-Path Particle Dosimetry). While standard tests indicated optimal aerodynamic properties (MMAD 3.03 μm), realistic modelling revealed a critical barrier. Both experimental and computational results showed excessive deposition in the upper parts of the airways, caused by a “Laryngeal Jet” that increases inertial impaction and turbulent dispersion. Standard Pharmacopoeial methods thus yield false-positive predictions of delivery efficiency for dyspnoeic patients. We propose a mechanistic hypothesis that rapid inhalation associated with air hunger filters out a significant portion of the therapeutic dose before it reaches the target tracheobronchial receptors, which may partially explain the inconsistent clinical outcomes. Clinical strategies must therefore shift from dose escalation to flow-governed delivery or shape-optimised carriers to overcome this aerodynamic filtration.

**Supplementary Information:**

The online version contains supplementary material available at 10.1038/s41598-026-55439-3.

## Introduction

Dyspnoea represents one of the most prevalent and distressing symptoms in internal medicine, significantly impacting patients’ quality of life. It constitutes a major source of global morbidity in subjects with advanced cardiovascular and respiratory diseases. Despite advances in palliative medicine, a safe and effective therapeutic strategy for refractory dyspnoea remains elusive^1,2^.

Dyspnoea is defined by the American Thoracic Society (ATS) as “a subjective experience of breathing discomfort that consists of qualitatively distinct sensations that vary in intensity”^2^. Unlike objective clinical signs, the subjective nature of dyspnoea means that the patient’s perceived distress does not necessarily correlate with measurable physiological parameters such as blood gas levels. Neurophysiologically, the symptom is postulated to arise from an “efferent-afferent mismatch”, i.e., a dissociation between the central motor command output to respiratory muscles and the incoming afferent feedback from pulmonary mechanoreceptors.

Inhaled furosemide (FUR) has emerged as a potential therapeutic candidate for the management of refractory dyspnoea. While primarily indicated for the oral and/or intravenous treatment of heart failure and oedema, evidence from case reports and randomised controlled trials newly suggests a beneficial effect of inhaled FUR on dyspnoea in both healthy volunteers and patients with chronic obstructive pulmonary disease (COPD)^3–5^.

Although inhaled FUR may induce mild bronchodilation by stabilising epithelial membranes^6^, the primary mechanism of action is hypothesised to be neurophysiological^4,5^. Inhaled FUR is believed to sensitise Pulmonary Stretch Receptors (PSRs), specifically the Slowly Adapting Receptors (SARs)^7^, localised within the smooth muscle of the conducting airways (generations 2–16). By lowering the firing threshold of these mechanoreceptors, inhaled FUR amplifies the afferent neural signal of lung inflation transmitted via the vagus nerve. This enhanced signalling mimics the feedback of a larger tidal volume, thereby reducing the abovementioned efferent-afferent mismatch and attenuating the perception of air hunger^8^.

Despite this physiological rationale, clinical efficacy data remain inconsistent. While some studies report significant relief, others utilising high-dose protocols failed to demonstrate a therapeutic effect^9^. We hypothesise that this heterogeneity might stem from inter-subject variability in aerosol deposition sites. Given that SARs are anatomically restricted to the tracheobronchial tree, successful delivery to this specific region is a prerequisite for drug efficacy. Consequently, alveolar deposition is therapeutically ineffective, while larger extrathoracic deposition represents a loss of the active pharmaceutical ingredient.

Notably, the majority of prior clinical studies failed to characterise the aerodynamic particle size distribution (APSD) or the regional deposition of the administered aerosol. Consequently, the lack of therapeutic response in some cohorts may be attributed to delivery failure rather than a lack of pharmacological efficacy. This issue highlights a broader challenge in respiratory drug development: the exceptionally high attrition rate of drugs during clinical trials^10^. This paper aims to demonstrate that advanced computational and experimental modelling represents a crucial tool to bridge the gap between standardised preclinical testing and clinical outcomes.

A critical limitation in the assessment of inhaled therapeutics is the reliance on standard Pharmacopoeial methods (Ph. Eur.)^11^, which employ standardised testing regimes and fail to replicate the dynamic breathing patterns of a dyspnoeic patient. Subjects experiencing severe dyspnoea exhibit elevated inspiratory flow rates and rapid acceleration. From a fluid dynamics perspective, inertial impaction is governed by the Stokes number (Stk), which is proportional to the flow velocity and the square of the particle diameter, $\:\mathrm{S}\mathrm{t}\mathrm{k}\propto\:U\cdot \:{d}^{2}$. Therefore, the pathological increase in inspiratory velocity directly elevates the Stk significantly increasing the probability of inertial deposition in the oropharynx. Furthermore, these high-flow conditions promote the formation of a “Laryngeal Jet”, a high-velocity turbulent flow structure that significantly enhances the inertial impaction and turbulent dispersion in the upper parts of the tracheobronchial tree^12^. During severe dyspnoea, peak velocities in this jet might range from 15 to 35 m/s, with the Reynolds number at the glottal constriction consistently reaching between 3,500 and 8,000^12^. This phenomenon creates a “Dyspnoea Paradox”, wherein the physiological manifestations of the symptom itself generate an aerodynamic barrier that impedes aerosol delivery to the target tracheobronchial region.

The objective of this study is to quantify aerodynamic barriers for inhaled medication using inhaled FUR as a model case. We combined standard Pharmacopoeial characterisation with in vitro measurements in a realistic human airway replica and Large Eddy Simulation (LES) to analyse regional deposition under simulated dyspnoeic breathing conditions. This approach allows for the assessment of whether therapeutic aerosol can effectively reach the SAR-rich lower tracheobronchial airways despite the aerodynamic challenges imposed by the pathological breathing pattern.

## Results

### Compatibility of the vibrating mesh nebuliser with the preparation

The compatibility study revealed a minimal difference in pH values between the commercial FUR Kabi 20 mg/2 mL product (8.20 ± 0.01) and the same FUR preparation after nebulisation (8.21 ± 0.01). The average FUR content after nebulisation was 101.4 ± 6.94% compared to the original commercial preparation; no decomposition products were observed in the High-Performance Liquid Chromatography (HPLC) chromatogram. These results confirmed that the Aerogen Solo vibrating mesh nebuliser (VMN) did not alter the chemical properties of the FUR preparation during the 20-minute nebulisation and is fully compatible with this medicinal product.

### Delivered dose

The dose of FUR delivered to the patient, determined using the standard Pharmacopoeial method, was 21.03 ± 2.92 mg. This represents 52.57 ± 7.31% of the initial dose filled into the nebuliser. This reduction is expected, as the breathing simulator alternates between inspiration and expiration, meaning the aerosol generated during the expiratory phase is not inhaled but vented. The total nebulisation time for 4 mL of FUR preparation (2 ampoules) ranged between 20 and 30.5 min, resulting in a mean dose delivery rate of 0.87 ± 0.35 mg·min⁻¹.

However, under the simulated dyspnoea breathing conditions (used in the in vitro deposition study described in “ Comparative lobar deposition in G4 and downwards (EXP vs. CFD vs. MPPD)”), the inhalation time is significantly shorter than during standard testing, despite the nebulisation rate remaining constant. This resulted in a lower effective delivered dose rate of 0.39 ± 0.15 mg·min⁻¹. This indicates that the delivered dose rate may decrease by more than 50% during realistic dyspnoeic breathing compared to standard testing conditions due to the significantly different I:E ratio.

### Aerodynamic particle size distribution

**Fig. 1:**
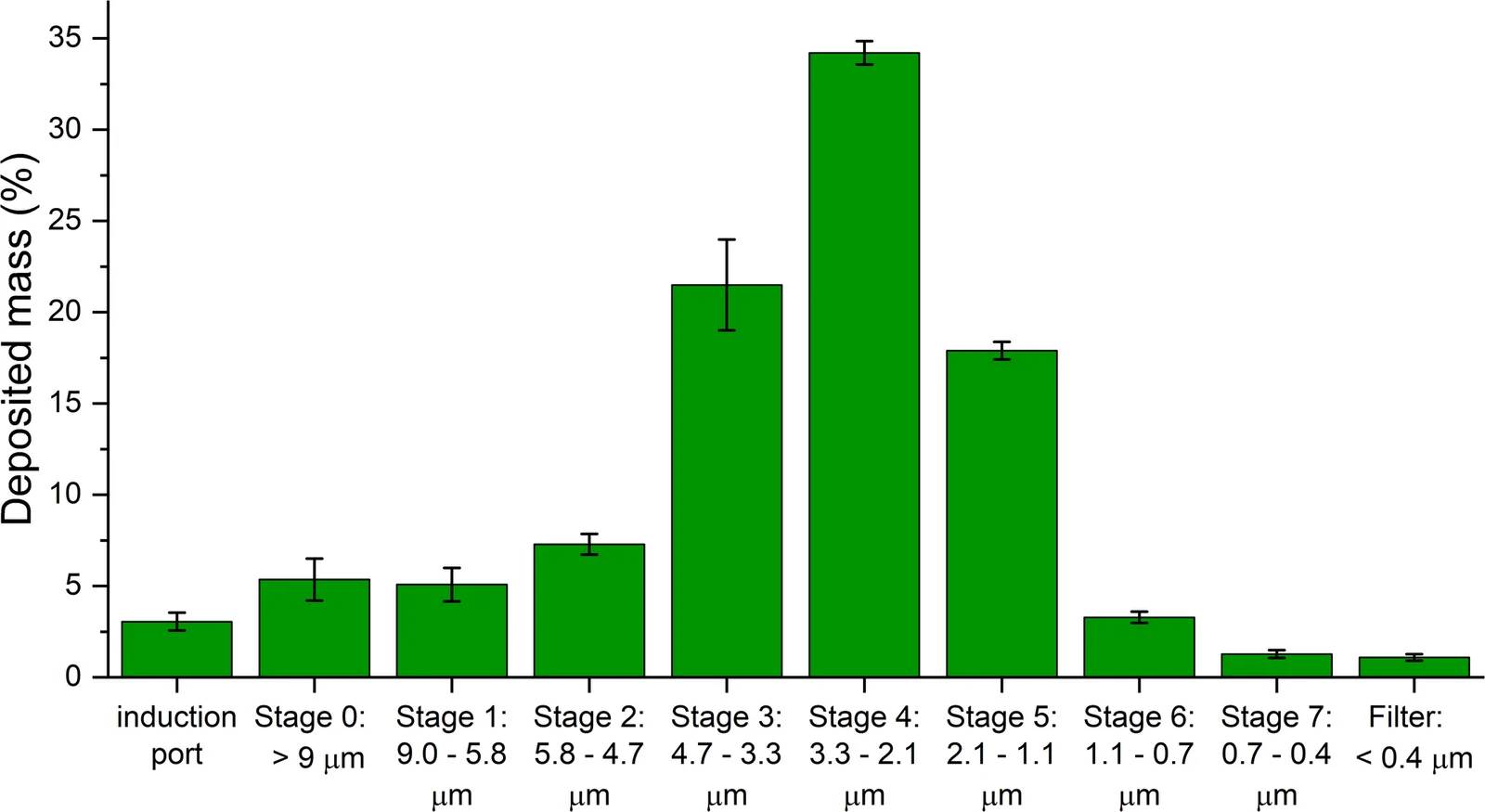
The mass aerodynamic particle size distribution of nebulised FUR preparation given by the Andersen cascade impactor with 7 stages (error bars represent standard deviations calculated from three repetitions).

The APSD, measured using the Andersen Cascade Impactor (ACI), demonstrated excellent repeatability (Fig. 1). The mass median aerodynamic diameter (MMAD) of the generated particles was 3.03 ± 0.03 μm. The geometric standard deviation (GSD) was 1.8 ± 0.01, and the estimated FPF was 81.2 ± 2.8%. These values indicate that the particles have the potential to effectively penetrate beyond the upper airways and deposit in the deeper parts of the lungs under standard resting breathing conditions. However, to obtain accurate information on regional deposition under pathological conditions, data from airway replicas or computational simulations are required (see Methods).

### MPPD modelling predictions

The probabilistic basis for comparison was provided by the Multiple-Path Particle Dosimetry (MPPD) Model (“Aerosol deposition in airway replica (experiment vs. CFD)”). Under the specific dyspnoeic boundary conditions (tidal volume 1390 mL, frequency 28.6 min⁻¹), the model predicted a Total Deposition Fraction of 62.3% for the entire respiratory tract. The regional deposition breakdown was as follows:

- Head (extrathoracic) deposition: 29.6%.
- Tracheobronchial (TB) deposition: 23.8%.
- Pulmonary (alveolar) deposition: 8.9%.

It is important to note that the MPPD simulation modelled a standard breathing cycle (inspiration followed by expiration), predicting that 37.7% of inhaled particles would be exhaled. This contrasts with the in vitro and Computational Fluid Dynamics (CFD) methodology, which utilised a breath-hold protocol to force sedimentation.

### Aerosol deposition in airway replica (experiment vs. CFD)

**Fig. 2:**
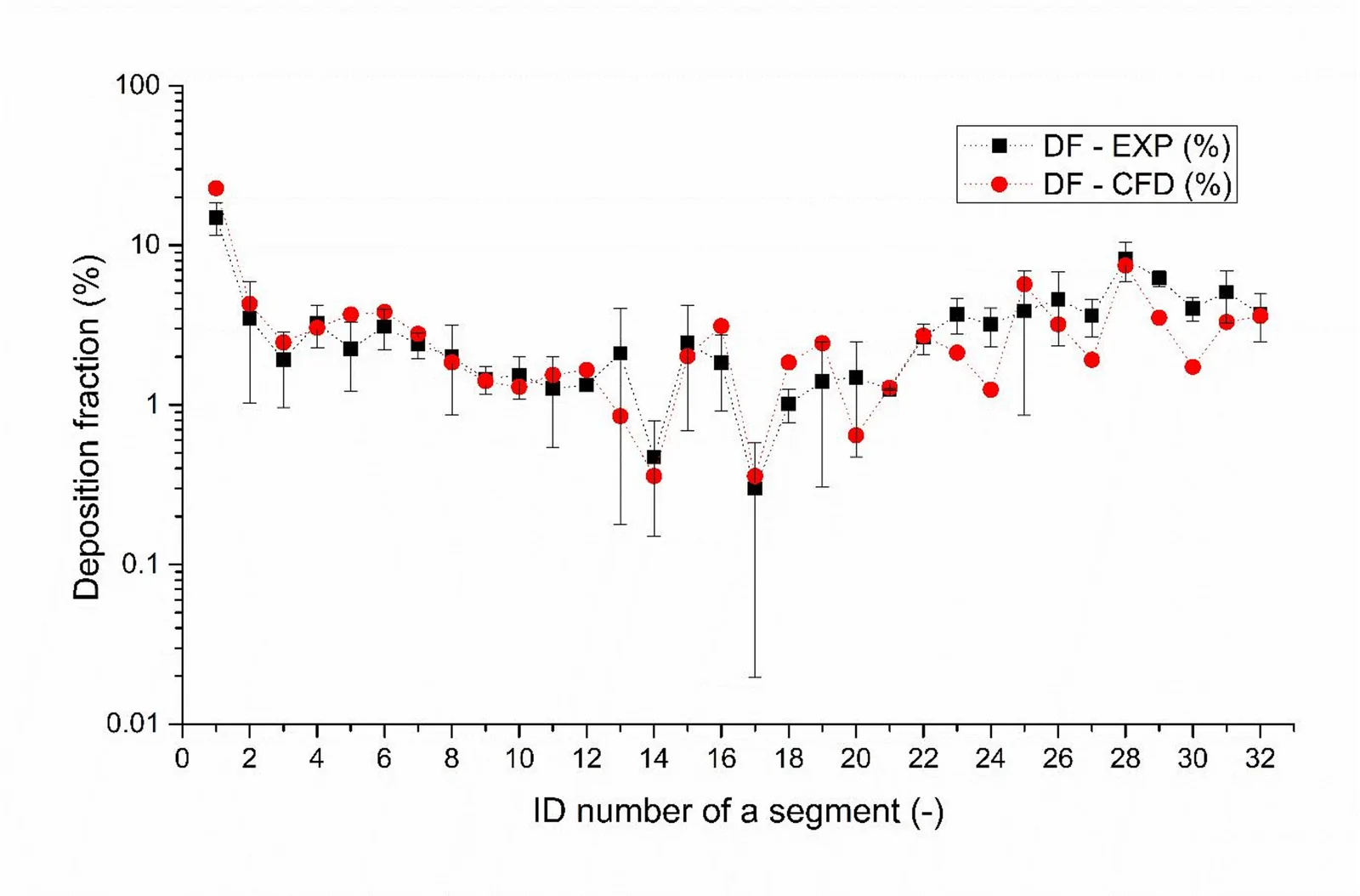
The comparison of deposition fraction in the airway replica obtained by experiment (EXP) and by CFD simulations. Error bars represent standard deviations.

The comparison of deposition fractions measured experimentally in the airway replica and using CFD LES is depicted in Fig. 2. Generally, good agreement was observed between the methods. The total deposition fraction in the replica (generations G0–G7) was 53.8% in the experiments and 66.2% in the LES simulations.

The highest discrepancy was observed in the upper airways. For example, deposition in the oral cavity (Segment 1) was 14.9% in the experiments compared to 22.8% in the simulations. The deposition fraction in the trachea (Segment 2) showed better agreement, with 3.5% detected in experiments versus 4.3% predicted by simulations. The total amount of deposited matter that penetrated beyond the upper airways (i.e., deposited in Segment 3 and downwards) represented 81.6% of the recovered dose in the experiments, compared to 72.9% in the simulations.

**Fig. 3:**
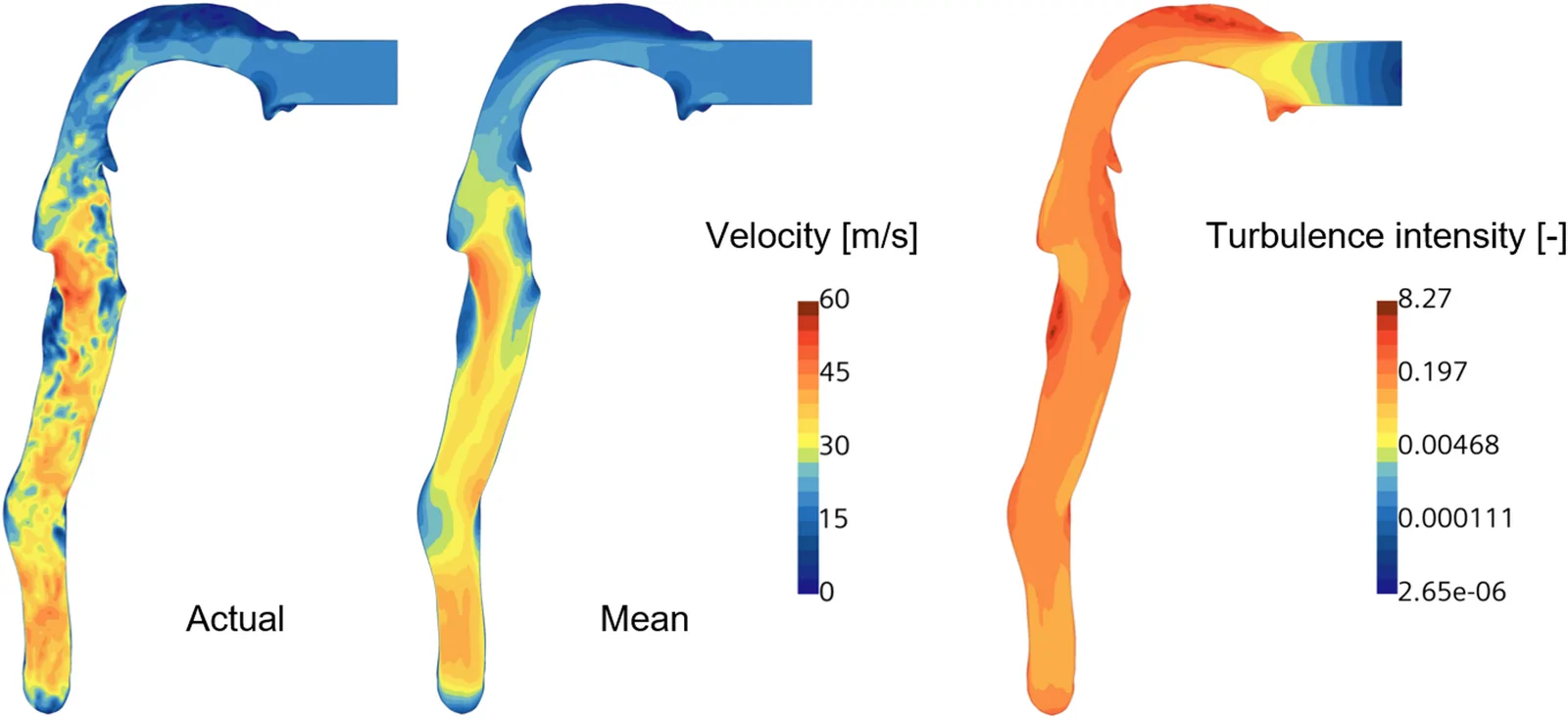
Computational fluid dynamics (LES) visualisation of the laryngeal jet at peak inspiratory flow. The panels compare the actual instantaneous velocity field (left), the time-averaged mean velocity field (middle), and the turbulence intensity (TI) propagating into the proximal tracheobronchial tree (right).

The significant particle deposition observed in the proximal airways is aerodynamically driven by the formation of a laryngeal jet. As visualised by the LES fluid dynamics results (Fig. 3), the high inspiratory flow rate generates a powerful, fully turbulent jet exiting the glottis. The actual instantaneous velocity field indicates the generation of eddies, while the time-averaged mean velocity field and the corresponding high turbulence intensity (TI) demonstrate how this flow structure propagates directly into the proximal tracheobronchial tree, promoting rapid inertial impaction before the aerosol can reach the deeper lung regions.

### Lobar deposition distribution

As shown in Table 1, the distribution of the deposition fraction (DF) was not strictly proportional to the distribution of airflow during inspiration. For instance, the Right Middle Lobe (RML) received only 6.2% of the deposition in the CFD simulations despite receiving 10% of the flow, whereas experimental results showed 10.7% deposition. Conversely, the Right Lower Lobe (RLL) showed excellent agreement between methods.

**Table 1:** The comparison of deposition fraction (DF) distribution in lobes obtained by experiment (EXP) and by CFD (LES) simulations.

Lobes (segments)	Flow rate share (%)	DF EXP (%)	DF-CFD (%)
LLL (6,14,15)	21	13.1	13.1
LUL (7, 13,16)	25	14.6	12.1
RUL (9, 19,21)	21	15.4	11.9
RML (11, 17,20)	10	10.7	6.2
RLL (12, 18,22)	23	16.9	17.2
Sum	100	70.7	60.6

###  Comparative lobar deposition in G4 and downwards (EXP vs. CFD vs. MPPD)

While a direct comparison of absolute total deposition is confounded by the breath-holding protocol used in the experiment versus the single inhalation in MPPD, the relative distribution of the deposited mass among the lung lobes provides interesting insight into the aerodynamic behaviour.

**Fig. 4:**
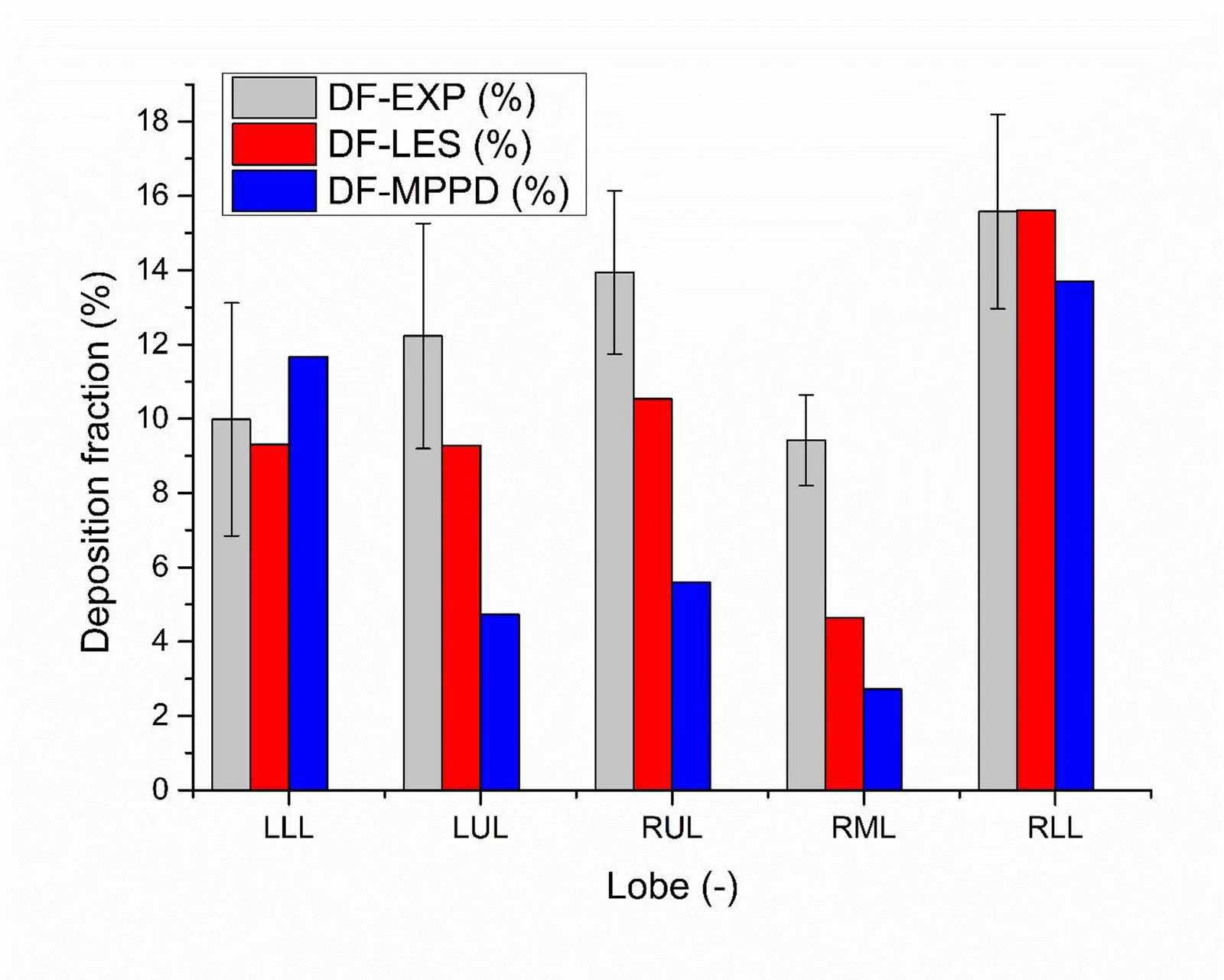
Comparison of the lobar deposition fraction distribution of particles obtained by experiment (EXP), and simulations by CFD (LES) and MPPD. Error bars represent standard deviations of experimental results.

To enable this comparison, the analysis focused on generations 4 and downwards, which corresponds to the anatomical definition of distinct lobes in the MPPD (Yeh and Schum)^13^ model. The MPPD data were postprocessed to account for the exhaled fraction, using the assumption that the airborne particles remaining after the first inhalation (i.e. predicted to be exhaled) would eventually deposit proportionally to the initial distribution during the breath-hold period (Fig. 4).

The results (Table 2) reveal a distinct disparity in the upper lobes. Both the experimental and CFD (LES) results show significantly higher deposition in the Left Upper Lobe (LUL) and Right Upper Lobe (RUL) compared to the MPPD prediction. For instance, in the RUL, the experimental deposition (13.9%) is more than double the MPPD prediction (5.6%). In contrast, the RLL shows remarkable agreement across all three methods. Note that MPPD in the standard Yeh and Schum^13^ model uses a different distribution of flowrates than our experimental and CFD case.

**Table 2:** The comparison of deposition fraction distribution in lobes (generations ≥ 4) obtained by experiment (EXP), CFD simulations, and MPPD modelling.

Lobe (segments)	Flow rate share (EXP and CFD) (%)		Flow rate share (MPPD) (%)	DF-EXP (%)	DF-CFD (%)	DF-MPPD (%)
LLL (14,15)	21		31	10.0	9.3	11.7
LUL (13,16)	25		16	12.2	9.3	4.7
RUL (19,21)	21		15	13.9	10.5	5.6
RML (17,20)	10		8	9.4	4.6	2.7
RLL (18,22)	23		31	15.6	15.6	13.7
Sum	100		100	61.1	49.4	38.4

**Fig. 5:**
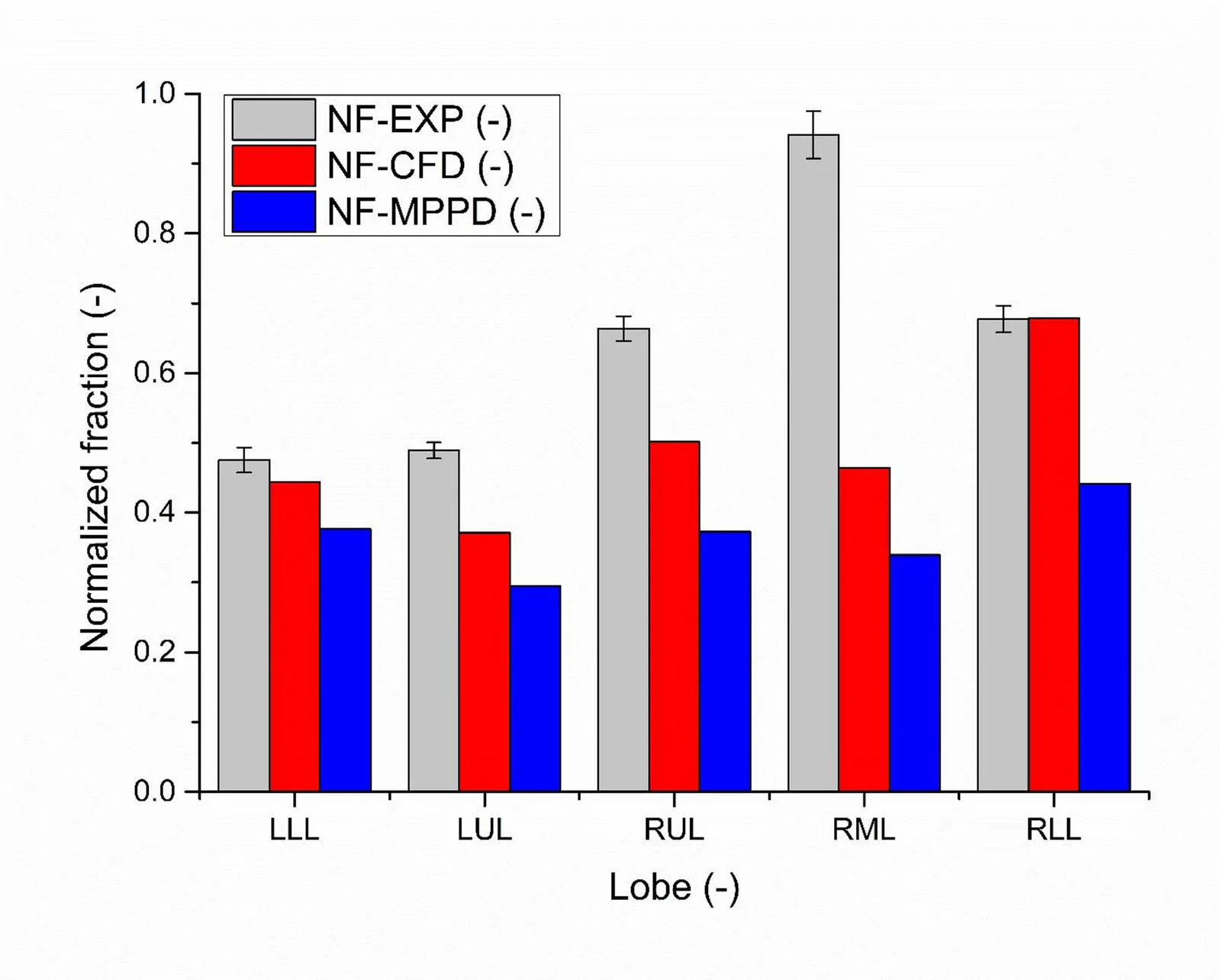
Normalised fraction (NF) across the lung lobes, comparing experimental data (NF-EXP), CFD simulation (NF-CFD), and the MPPD model (NF-MPPD). The values represent the ratio of regional deposition fractions to the respective lobar flow rate shares. Error bars indicate standard deviations calculated from the experimental data across three repetitions.

To explicitly quantify the divergence between the volumetric distribution assumed by 1D models and the inertia-driven distribution captured by 3D models, we calculated a Normalised Fraction (NF) for each lobe. This metric is defined as the ratio between the regional DF and the corresponding lobar Flow Rate Share. The comparison of the NF across the experimental, CFD, and MPPD methods is visualised in Fig. 5. This metric explicitly illustrates whether a lobe receives a proportionate mass of aerosol relative to its ventilation, demonstrating the differences between the predictive models.

## Discussion

For successful drug delivery to the lungs, achieving an optimal particle size is a prerequisite. The optimum aerodynamic particle size range between 0.5 and 5 μm^14^ is readily provided by modern application devices, such as the VMN used in this study^15^. However, even with an appropriate particle size distribution, the active substance may not reach the deeper parts of the respiratory tract, because inhalation drug delivery is conditioned by the synchronisation of breathing and aerosol formation^16^. Hence, detailed studies should always be performed to quantify drug losses in the upper respiratory tract and to determine the correct effective dose^17^ before launching any inhalation system to clinical trials. This study combined in vitro measurements and numerical modelling to investigate these losses under specific dyspnoeic conditions.

A primary objective of this study was to evaluate the predictive power of different modelling approaches for inhaled furosemide delivery under dyspnoeic conditions. It is crucial to note that a direct comparison of absolute total deposition fractions between the physical replica and the MPPD model (Yeh and Schum^13^ is constrained by their geometric and methodological differences. The MPPD model simulated the entire respiratory tract (up to the alveoli) under a standard breathing cycle. In contrast, the physical and CFD models were anatomically truncated at the 7th generation and utilised a breath-holding protocol.

The comparison between experimental data and CFD simulations (Fig. 2) showed generally good agreement, although CFD slightly overpredicted deposition in the extrathoracic regions (66.2% vs. 53.8%). This overprediction of CFD-determined deposition in the upper airways has been observed in other studies. Koullapis et al.^18^ suggested that one reason can be the modelling of turbulence in the near-wall region, which can be mitigated by implementing corrections to velocity fluctuations^19^. From an experimental perspective, the large airways (oral cavity, trachea) pose a challenge for rinsing; these segments are too large to be immersed in liquid and must be rinsed manually, potentially introducing minor variability.

While one-dimensional empirical dosimetry models like MPPD provide a highly convenient and rapid tool that successfully predicts overall total lung deposition trends, our results demonstrate that they significantly diverge from 3D LES and experimental data regarding regional lobar distribution. When comparing the relative lobar particle distribution within the conducting airways (generations 4–7), significant mechanistic differences emerge. The MPPD model, which relies on idealised branching, significantly underestimates deposition in the upper lobes compared to both the experiment and the 3D CFD simulations (Table 2). This discrepancy arises because standard MPPD simulations, utilising Yeh and Schum^13^ morphometry, distribute the inhaled airflow—and therefore the aerosol particles—primarily based on the downstream volumetric capacity of the lobes, assuming uniform lung expansion. As a 1D model, MPPD inherently lacks the physical mechanics of 3D inertia; it cannot capture complex aerodynamic phenomena such as the asymmetric skew of the high-velocity laryngeal jet. Consequently, MPPD severely underestimates the formation of localised upper-lobe hotspots caused by high-velocity impaction at proximal bifurcations. For targeted therapies like furosemide, where identifying and avoiding localised receptor saturation is paramount, relying solely on 1D MPPD predictions is insufficient.

In reality, as captured by our LES and in vitro models, the high momentum of the jet forcibly directs a disproportionately large mass of particles straight down the trachea, causing them to impact on the airway wall and creating localised deposition “hotspots”. While previous studies, such as the subject-specific analysis by Kuprat et al.^20^, have demonstrated that ventilation is often heterogeneous and not strictly proportional to Functional Residual Capacity (FRC) volume (e.g., the RUL accounted for 33% of the lung volume but received only 20% of the flow), our data explicitly highlight the dominance of inertial dynamics. The jet essentially drives particles in its direction and modifies their distribution. Therefore, while MPPD remains a valuable tool for general macroscopic dosimetry, its use is limited and insufficient in targeted therapies, such as furosemide administration, where identifying and avoiding localised receptor saturation at proximal bifurcations is paramount.

Based on our experimental data, we propose a mechanistic hypothesis that may partially explain the inconsistent clinical efficacy of inhaled furosemide observed in previous trials^9,21^, alongside other pharmacokinetic factors. The results reveal that under dyspnoeic breathing conditions (mean inspiratory flow ~ 3.3 L·s⁻¹), the upper airways act as a highly efficient filter, capturing 20 to 30% of the administered dose. We identify this phenomenon as the “Dyspnoea Paradox”. The physiological state of air hunger drives the patient to breathe with high velocity and rapid acceleration. This significantly enhances the inertial deposition in the upper airways and also creates a powerful laryngeal jet (as shown in Fig. 3), a turbulent flow structure extending from the trachea to the upper parts of the bronchial tree. This jet increases inertial impaction and turbulent dispersion not just in the larynx (where MPPD predicted ~ 30% deposition), but also in the proximal bronchial generations. Consequently, the very symptom requiring treatment (dyspnoea) generates an aerodynamic barrier that filters out a significant portion of the therapeutic aerosol before it can reach the distal conducting airways.

The neurophysiological hypothesis posits that FUR efficacy depends on sensitising SARs located in the smooth muscle of the tracheobronchial tree (generations G2 – G16)^7^. While SARs are distributed throughout these generations, the therapeutic contribution of the proximal airways (G2–G7) is limited by two factors: their relatively small surface area and the phenomenon of localised saturation. Under dyspnoeic flow conditions, inertial impaction concentrates the drug into specific ‘hotspots’ (bifurcations) within G2–G7. This likely leads to receptor saturation in these focal areas, while failing to utilise the extensive surface area of the distal conducting airways (G8–G16), where the drug could recruit a significantly larger population of SARs. Thus, generations G8–G16 represent the accessible and most effective target region. Hence, our data challenge the traditional metric of Total Lung Dose. For this specific therapy:

- Alveolar deposition is pharmacologically ineffective as SARs are absent in the respiratory zone.
- Oropharyngeal deposition represents a significant loss of drug due to the “Dyspnoea Paradox”.
- Distal tracheobronchial deposition (generations G8 – G16) represents the true target region, which is difficult to reach under high-flow conditions.

The lack of a linear dose-response relationship in clinical studies can be attributed to aerodynamic saturation. Increasing the nebuliser charge under dyspnoeic flow conditions likely increases the mass impacting the upper parts of the airways without sufficiently increasing the dose delivered to the distal airways, where SAR density is high.

This study highlights the inadequacy of standard Pharmacopoeial measurements for predicting clinical outcomes in dyspnoeic patients. The ACI data indicated an MMAD of 3.03 μm and an FPF of > 80%. Under standard testing conditions (28.3 L·min⁻¹, laminar flow), these values imply excellent lung delivery. However, our realistic modelling shows that the transition to the high-flow, turbulent regime of dyspnoea proportionally magnifies the Stokes number of the aerosol droplets. Consequently, even these “optimal” 3 μm particles possess sufficient momentum to shift the primary deposition mechanism to proximal inertial impaction in the throat and primary bronchi. The standard methods, therefore, generate a false positive prediction of delivery efficiency by failing to replicate the patient’s pathological breathing pattern.

Our results show that the Aerogen Solo VMN is fully compatible with the commercial FUR formulation and does not affect its stability. Therefore, the bottleneck is not the chemical composition, but the aerodynamics of administration.

For clinical practice, this implies that simple dose escalation is an ineffective strategy. To overcome the laryngeal jet and bypass the proximal impact zones, delivery strategies must address the increased inertial forces. In the context of current liquid nebulisation therapy, optimisation is limited to flow-governed delivery (e.g., breath-actuated nebulisers releasing aerosol only during the low-flow phase or coached breathing manoeuvres) and reducing the droplet size to compensate for the elevated Stokes number. However, liquid droplets are inherently spherical due to surface tension. To further exploit aerodynamic benefits, future therapeutic development could shift towards solid-state formulations, where anisotropic particle shapes (e.g., microrods) or magnetic guidance can be engineered to bypass the laryngeal filter^22,23^.

The development of inhaled therapeutics is a complex and resource-intensive process, estimated to cost over $1.5 billion and taking approximately 12 years from discovery to market entry^24^. Notably, the attrition rate is particularly severe for respiratory drugs, with a cumulative probability of reaching the market of only 3%, compared to 6–14% for other disease areas^10^. Our findings suggest that this high failure rate may be partially attributed to the reliance on inadequate preclinical testing methods (such as standard ACI or the Next Generation Impactor) that fail to detect aerodynamic barriers like the laryngeal jet. If a drug shows excellent fine particle fraction in vitro but fails in clinical trials due to throat deposition (as observed with high-dose furosemide^9)^, the cost of failure is immense. Implementing realistic aerodynamic modelling—combining physical replicas and CFD—early in the development pipeline offers a crucial tool to bridge this gap, potentially identifying ineffective delivery systems before they enter costly clinical phases.

Several limitations of this study must be acknowledged. First, the study utilised a realistic but rigid geometry representing a single healthy subject. The chosen replica, combining the Cast A oral cavity^25^ and the Schmidt et al. digital reference model^26^, is a standardised and previously validated geometry utilised, e.g. as the SimInhale benchmark^18^. It allows for direct comparison with a vast body of existing literature^27,28,18,29^; nonetheless, it is still a single airway geometry and hence inherently limits the generalisability of absolute deposition values across a heterogeneous patient population. The human respiratory tract exhibits profound inter-subject anatomical variability, which can significantly shift localised aerosol deposition patterns. Consequently, this single-subject model does not account for morphological variances or the dynamic airway compliance observed in vivo. During the rapid inspiration characteristic of dyspnoea, dynamic airway collapse or disease-induced airway remodelling (such as bronchoconstriction or mucus hypersecretion) could narrow the flow path. This would increase local flow velocities even further, making the inertial impaction and the “Dyspnoea Paradox” effect observed in our baseline healthy model even more pronounced. To elevate the predictive power of computational dosimetry from the individual to the population level, future studies should incorporate multi-subject cohorts or Statistical Shape Models (SSMs)^20^ to systematically quantify how specific morphological deviations influence aerodynamic filtration.

Furthermore, to maintain consistency with standard Pharmacopoeial methods (ACI), the experiments were conducted at 5 °C without simulating evaporation or condensation. In a clinical setting, the warm and humid environment of the lungs (37 °C and 100% relative humidity) could induce hygroscopic growth of the droplets. This increase in particle size would likely further enhance inertial impaction, thereby reinforcing the “Dyspnoea Paradox” filtering effect described in this study.

It should also be noted that the 3D models (experiment and CFD) were anatomically truncated at the 7th generation. Therefore, deposition in the target region (G8–G16) was not directly measured but inferred from the fraction passing through the outlets or modelled using 1D dosimetry (MPPD), which lacks the resolution of 3D flow structures. Finally, the LES simulation slightly overpredicted deposition compared to the experiment (66.2% vs. 53.8%). This discrepancy is likely attributed to the “trap” boundary condition, which neglects droplet bounce or splashing on the wet airway surface, and the breath-hold protocol used to force sedimentation, which does not fully replicate the exhalation phase of a respiratory cycle.

Finally, it is important to emphasise that aerosol deposition is only the first phase of drug delivery. For furosemide to be therapeutically active, the deposited particles must overcome significant post-deposition biological barriers to reach the SARs located within the smooth muscle layer. The conducting airways are lined with a viscoelastic mucus layer, composed predominantly of water and extensively cross-linked mucin glycoproteins (such as MUC5AC and MUC5B)^30,31^. When particles or droplets land on the mucosal surface, their entrapment and subsequent penetration are governed by electrostatic interactions and the steric hindrance of the mucin mesh^32,33^.

Because the airway mucus possesses a strong negative charge, it strongly interacts with charged particulates. In clinical practice, furosemide is administered as a mildly buffered alkaline solution (pH > 8.0) to maintain its solubility. However, high-velocity inertial impaction in proximal hotspots—as identified in our aerodynamic models—may dump a large mass of the drug into a minuscule surface area. Once deposited, the limited volume and buffering capacity of the local epithelial lining fluid may be overwhelmed, potentially altering the local pH and causing the drug to precipitate. The undissolved drug fraction cannot penetrate the epithelial barrier and is rapidly subjected to mucociliary clearance, propelling it cephalad before it can exert any pharmacological effect. Simultaneously, the limited number of SARs in these highly localised proximal areas may reach rapid saturation, while the vast, therapeutically vital populations of SARs in the distal conducting airways (G8–G16) remain completely unexposed due to the upstream aerodynamic filtration. Although explicitly modelling these human-specific lung microenvironments and dissolution kinetics utilising small-scale culture systems or Physiologically Based Pharmacokinetic (PBPK) models is beyond the scope of this aerodynamic study, our highly resolved regional deposition data provide the essential input boundary conditions required for such future translational research.

This study integrated experimental cascade impaction, realistic in vitro airway replication, and advanced computational modelling to characterise the delivery of inhaled furosemide under conditions of severe dyspnoea. Based on the results, we draw the following conclusions:

1. Formulation suitability: the Aerogen Solo vibrating mesh nebuliser is chemically compatible with the alkaline furosemide preparation, delivering a consistent aerosol with a high fine particle fraction (> 80%) and maintaining the stability of the active substance.
2. The “Dyspnoea Paradox”: we quantified the aerodynamic barrier created by the patient’s condition. High inspiratory flow rates typical of dyspnoea cause a significant increase in the inertia of inhaled particles. Our realistic models (experiment and LES) demonstrated that this phenomenon transforms the upper airways into a highly efficient filter, capturing a significant portion of the dose before it can reach the lower airways.
3. Targeting efficiency: the neurophysiological target for dyspnoea relief, the SARs, resides primarily in the smooth muscle of the tracheobronchial tree (generations 8–16). Our data suggest that under uncontrolled dyspnoeic breathing, the target region (distal conducting airways) is difficult to reach due to substantial losses in the oropharynx and main bronchi, while alveolar deposition represents pharmacologically ineffective waste.
4. Methodological implications: standard Pharmacopoeial measurements (Ph. Eur.) performed at constant low flow rates fail to capture these inertial effects, leading to a “false positive” prediction of delivery efficiency. Furthermore, 1D dosimetry models (like MPPD) may underestimate localised deposition hotspots in the upper lobes caused by the 3D complexity of the laryngeal jet.
5. Clinical recommendation: the observed lack of linear dose-response in clinical trials may be partially explained by aerodynamic saturation, rather than solely pharmacological inefficacy. Future clinical strategies should not focus merely on increasing the nominal dose, but on flow-governed delivery strategies. Implementing breath-actuated nebulisers, coached breathing manoeuvres, or size and shape optimised particles to reduce peak inspiratory flow is essential to bypass the laryngeal filter and maximise engagement with SARs.

This multi-modal modelling approach provides a mechanistic explanation for previous clinical failures and offers a robust platform for optimising future inhaled therapies for refractory dyspnoea.

## Materials and methods

Three methodological approaches were employed to characterise the properties of the inhalation system and assess the deposition and transport of inhaled particles within human airways. Following the core hypothesis of this study, a series of experiments using the Aerogen Solo VMN and an aqueous solution of FUR were conducted. First, the delivered dose and APSD according to the methods described in Ph. Eur. (2.9.44) were assessed. This was followed by the calculation of the dose delivered to various regions of the airways using the MPPD model. Subsequently, in vitro deposition measurements using a physical replica of the human respiratory tract (male geometry, oral cavity to the 7th generation of branching) and a programmable breathing simulator were performed. Finally, computational simulation of the inhalation within the digital twin of the airway replica was performed. These experimental and computational methods were compared to assess their potential to realistically estimate the local deposited dose and provide caretakers with better information about the behaviour of the inhalation system in real patients.

### Nebuliser selection and confirmation of FUR compatibility

Patients suffering from respiratory diseases need fast and efficient therapy, particularly in acute cases. This need is generally fulfilled by inhalation of medication in the form of aerosols administered to the lungs using a suitable device, such as a nebuliser. Among the available nebuliser types, VMNs, which produce an aerosol by extruding the medication through a vibrating mesh, represent the preferred choice^34^. VMNs have several advantages, including a low residual volume, high nebulisation rate, and precise particle size definition governed by the mesh aperture, ensuring a high fine-particle fraction.

FUR substance was obtained from Fagron, Czech Republic. Water for injection (WFI) was used as a solvent throughout the whole study. All samples were always protected from light during manipulation and storage. Nebulisation was performed using the VMN Aerogen Solo (Aerogen Ltd., Galway, Ireland). To assess the compatibility of the VMN with the FUR solution, 4 mL of the commercial aqueous injection preparation FUR Kabi 20 mg/2mL (2 ampoules) were poured into the Aerogen Solo nebuliser chamber.

**Fig. 6:**
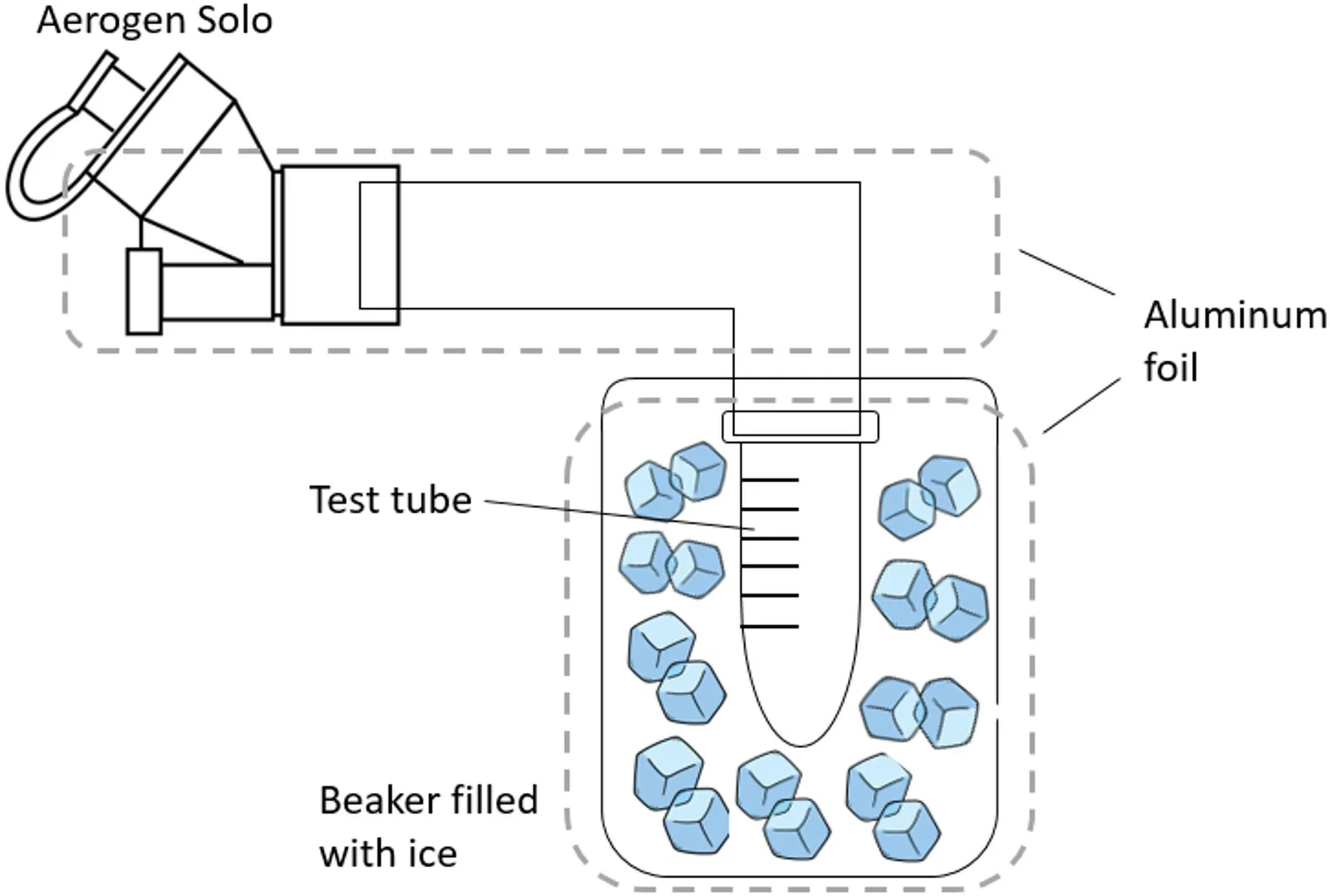
Experimental setup for confirmation of FUR compatibility with VMN.

The produced aerosol was collected in a cooled glass tube covered with aluminium foil (Fig. 6) for 20 min (the expected time of inhalation therapy). The FUR content was estimated by HPLC. The pH value of the solution before and after the nebulisation test was measured using a pH-meter Sentron SI400 with a MicroFET pH electrode (Sentron Europe B.V., Leek, Netherlands). All nebulisation tests were performed in triplicate, and the results are expressed as the mean ± standard deviation (SD). These results were compared with the FUR content and pH value of the commercial preparation to ensure that no deviations of physical and chemical properties took place during the laboratory procedures.

### Reagents and analytical method

FUR Impurity A (FUR A) was obtained from the Council of Europe – EDQM, Strasbourg, France. Methanol CHROMASOLV^®^ gradient grade, acetonitrile (ACN) CHROMASOLV^®^ gradient grade, formic acid 95%, and triethylamine (TEA) 99.5% were obtained from Sigma-Aldrich, Czech Republic. 18 MΩ.cm ultrapure water was obtained from a Milli-Q^®^ Integral water purification system with a 0.22 μm Millipack^®^ output filter (Millipore, USA).

The chromatographic analysis was used for the determination of FUR in the samples; FUR A was used to detect chemical degradation. A HPLC system Liquid Chromatograph - Nexera X2 (Shimadzu, Kyoto, Japan) with online detection using a diode array detector (SPD-M30 A) was used. The chromatographic system consisted of two quaternary pumps (LC-30 AD), a degasser (DGU-20A5R), an autosampler (SIL-30AC), a column oven (CTO-20AC), a communication bus model (CBM-20 A), and computer software (LabSolution). A reversed-phase LiChrospher column, 5 μm, C18 100 Å, 125 × 4 mm (Phenomenex, USA) was used for the separation.

The mobile phase was prepared by mixing the buffer solution and ACN in a ratio of 75:25 (v/v) and adjusted to a pH of 5.75. The buffer solution was prepared by mixing 200 mL of water, 50 µL of formic acid, and 150 µL of TEA. Before use, the aqueous phase was filtered through a 0.22 μm nylon membrane filter. Analytes were eluted using a flow rate of 1.5 mL·min⁻¹. A volume of 10 µL of the standard and sample solutions was injected every 2.5 min; the analytes were detected at 270 nm. A standard solution was prepared by dissolving the substances in methanol to reach the final concentrations of 50 µg/mL for FUR and 10 µg/mL for FUR A. The standard solution was protected from light and stored at 4 °C in the dark for a maximum of 24 h.

The analytical method was validated according to ICH Q2 (R2) guidelines (ICH Expert Working Group 2005). The system suitability (i.e., repeatability of retention times and areas, number of theoretical plates, resolution, tailing factor), precision, and linearity were evaluated during method validation as described by^35^. All sample solutions were stored in amber glass bottles (protected from light) and analysed directly without any dilution; each sample was measured in triplicate.

### Delivered dose

**Fig. 7:**
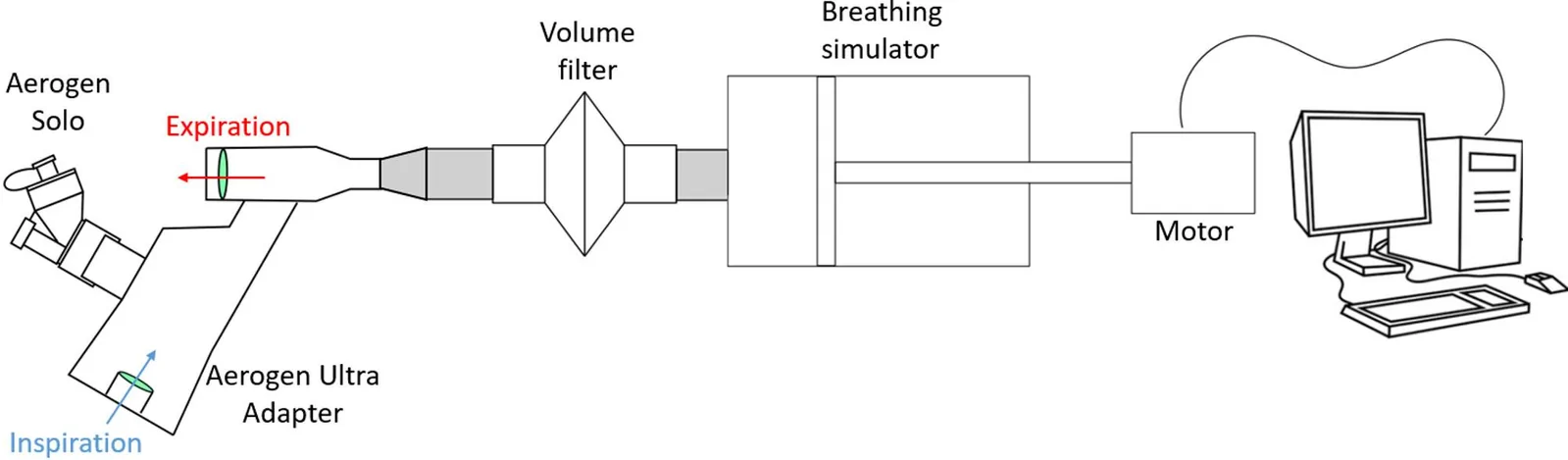
Measurement of the delivered dose.

The delivered dose and delivered dose rate of nebulised FUR were assessed according to the methods described in Ph. Eur. (2.9.44). The Aerogen Solo VMN was attached to an Aerogen Ultra adapter connected to a low-pressure and low-volume filter (Clear-Guard 3 Breathing Filter, Intersurgical Ltd., Wokingham, United Kingdom), which was further connected to a breathing simulator (Fig. 7). The adapter was equipped with one-way flap valves to alternate airflow between inspiration and expiration. The nebuliser was filled with 4 mL of the commercial FUR Kabi 20 mg/2 mL solution (2 ampoules).

The breathing simulator operated with a sinusoidal breathing pattern, a tidal volume of 500 mL, and a breathing frequency of 15 min⁻¹, in agreement with Ph. Eur. The production of aerosol began concurrently with the simulated breathing process. After a one-minute nebulisation interval, the apparatus filter was replaced. This process continued until the entire dose in the nebuliser was aerosolised. The delivered dose measurements were performed in triplicate, and the data are presented as the mean ± SD.

### Aerodynamic particle size distribution

An ACI was used to analyse the aerodynamic particle size distribution. The ACI was cooled to 5 °C to prevent particle evaporation, as recommended by Ph. Eur. (2.9.44). A custom-moulded adapter for the Aerogen Ultra mouthpiece was placed at the inlet of the ACI induction port. The outlet of the ACI was connected to a vacuum pump through a TSI 4040 flow meter (TSI Inc., Minnesota, USA). The Aerogen Solo nebuliser was connected to the assembly through the Aerogen Ultra adapter. The sampling duration was one minute at a flow rate of 28.3 L·min⁻¹. After sampling, each stage of the ACI was washed with methanol to extract the deposited FUR; the samples were analysed by HPLC. The fine particle fraction (FPF), MMAD, and GSD were calculated from the measured FUR mass in each ACI stage. The cascade impactor experiments were conducted in triplicate, with the results evaluated and reported as the mean ± SD.

### Multiple-path particle dosimetry modelling

To provide a probabilistic assessment of regional aerosol deposition, the MPPD model (Version 3.04, Applied Research Associates, Inc., Raleigh, NC, USA) was employed. This model calculates the deposition probability of particles using a mechanistic approach based on the single-path and multiple-path algorithms described by^36,37^. It enables rapid estimation of deposited dose in human and animal airways under various breathing conditions, although it relies on empirical efficiency functions rather than solving the full three-dimensional (3D) flow field.

The simulation was configured using the “Human (Limited)” lung geometry, which utilises the Yeh and Schum 5-Lobe model^13^ to account for lobar asymmetry. The FRC was scaled to 3300 mL (standard adult male). The input parameters were matched to the experimental boundary conditions as follows:

- Aerosol Properties: MMAD of 3.03 μm and GSD of 1.80.
- Breathing Parameters: To simulate the dyspnoea breathing pattern used in the in vitro and CFD studies, the model was set to oral breathing with a tidal volume of 1390 mL and a breathing frequency of 28.6 min⁻¹^38^. The inspiratory fraction was set to 0.2 (inhalation time 0.42 s), resulting in a mean inspiratory flow rate of approximately 3.3 L·s⁻¹.
- The inhalability adjustment was enabled to account for the aspiration efficiency at the mouth during high-flow oral breathing.

###  Aerosol deposition in airway replica

The deposition measurements were conducted using a pre-existing realistic model of human respiratory airways, the development and dimensions of which were thoroughly described by Lizal et al.^27^. The model was created by combining multiple geometries and included airways from the oral cavity extending to the 7th generation of tracheobronchial branching (generations G0–G7). The geometry of the oral cavity was originally obtained by the Lovelace Respiratory Research Institute (Albuquerque, NM, USA) and referred to as ‘cast A’ in previous literature^25^. The tracheobronchial tree was derived from the digital reference model of Schmidt et al.^26^, which was based on CT scans from a cadaver representing a healthy Caucasian male. No human participants were recruited, and no new human tissues or data were collected specifically for the present study. Therefore, according to institutional and national guidelines, formal ethical approval and informed consent were not required. To facilitate the numerical simulations and experiments, cylindrical inlet and funnel-like outlet segments were added to allow the prescription of boundary conditions or the connection of tubing.

**Fig. 8:**
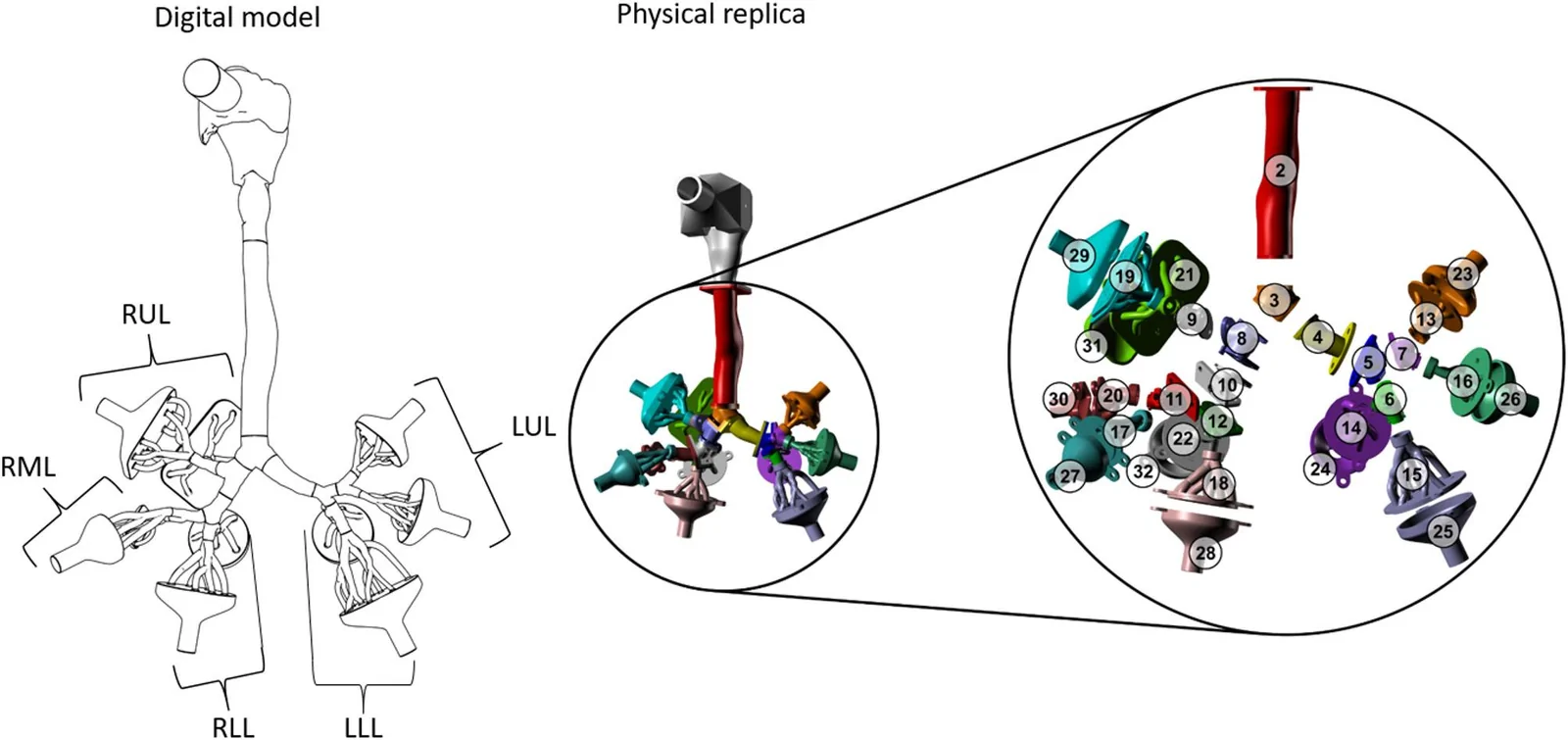
The schematic of the digital model (left) and experimental replica (right). The model is segmented and has two outlets per lobe; LUL denotes the left upper lobe, LLL denotes the left lower lobe, RUL denotes the right upper lobe, RML denotes the right middle lobe, and RLL denotes the right lower lobe.

The resulting digital model has ten outlets, with two in each lobe, and was physically segmented into key geometric airway parts, such as the trachea or the first bifurcation (Fig. 8). To ensure clarity across the different modelling approaches, specific terminology is used consistently throughout this study. The airway replica is divided into discrete *segments* based on manufacturing requirements and branching topology. These physical *segments* are numbered from top to bottom (1–22), with the oral cavity designated as Segment 1, the trachea as Segment 2, and so on (see Fig. 8). These segments correspond to anatomical airway *generations* (G) according to the Weibel model^39^. While the segments represent the building blocks of the physical and computational domain, the generations denote the depth within the tracheobronchial tree (G0–G7). Finally, the term *stage* is reserved exclusively for the *stages* of the ACI, corresponding to specific aerodynamic particle size fractions (Fig. 1).

For the purpose of deposition experiments, a 3 mm-thick shell was created around the digital geometry. Flanges with screws or bayonet joints were integrated between the segments to enable model assembly. The segments were fabricated on a Stratasys J850 printer (Stratasys, Minnesota, USA) using VeroClear material with an accuracy of 0.015 mm and a layer thickness of 14 μm. Prior to each experiment, the assembled replica was leak-tested, and any detected leaks were sealed using silicone sealant.

**Fig. 9:**
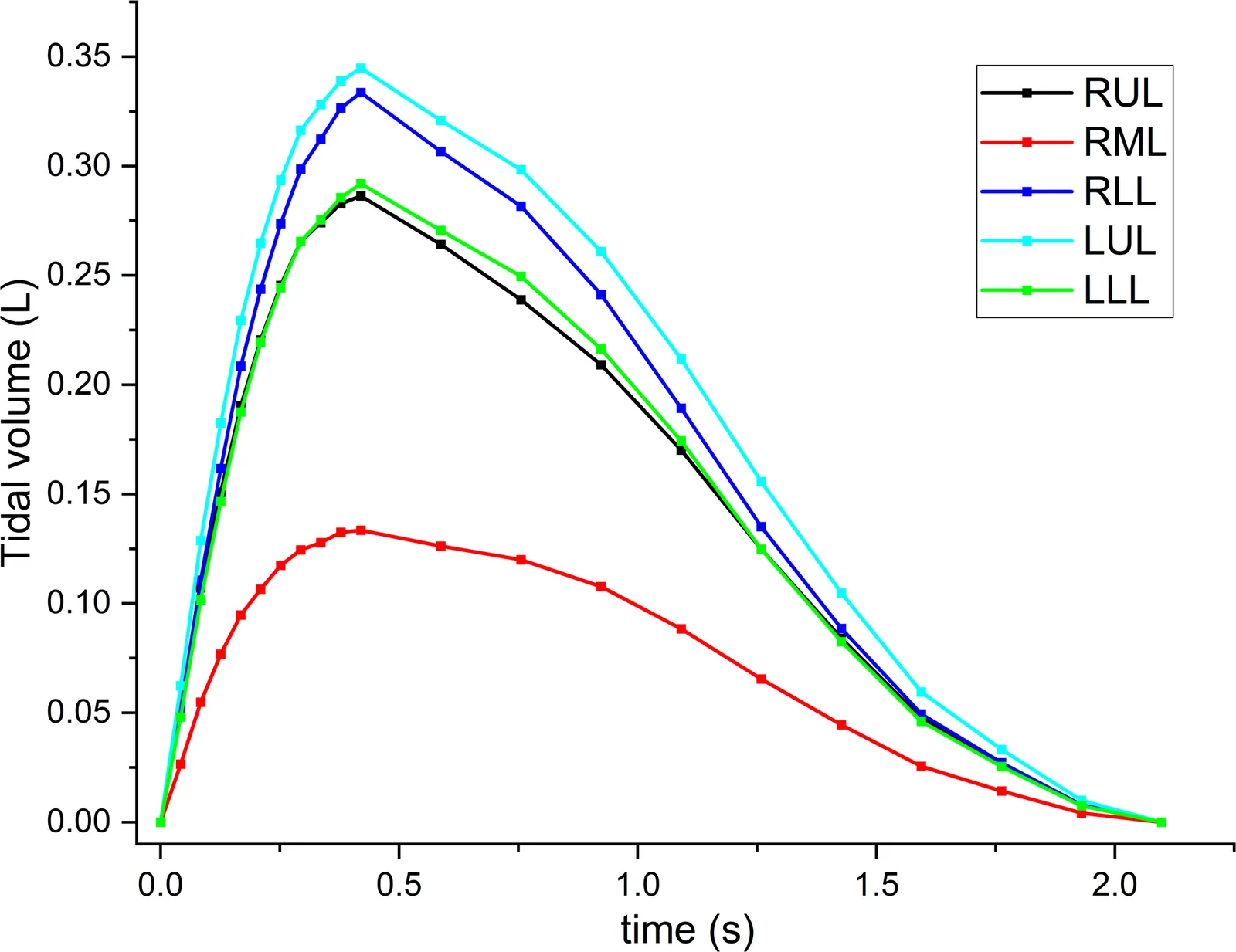
The reconstructed lobar breathing profiles of a dyspnoea patient.

The breathing parameters used in this study were based on the work of O’Donnell et al. (2009), who reported the breathing patterns of COPD patients during dyspnoea. The specific parameters included a breathing frequency of 28.6 min⁻¹, a tidal volume of 1.39 L, and an inspiratory-to-expiratory (I:E) ratio of 1:4. The breathing profile (Fig. 9) was reconstructed based on the shape of the breathing cycle and the flow rate distribution between the lung lobes measured and reported by^40^. The breathing simulator used in the experimental part of the study consisted of five pistons with programmable movement. Each piston corresponded to one lung lobe and moved according to the defined profile. Consequently, the ten outlets of the airway replica (two per lobe) were connected to the corresponding five pistons of the simulator.

**Fig. 10:**
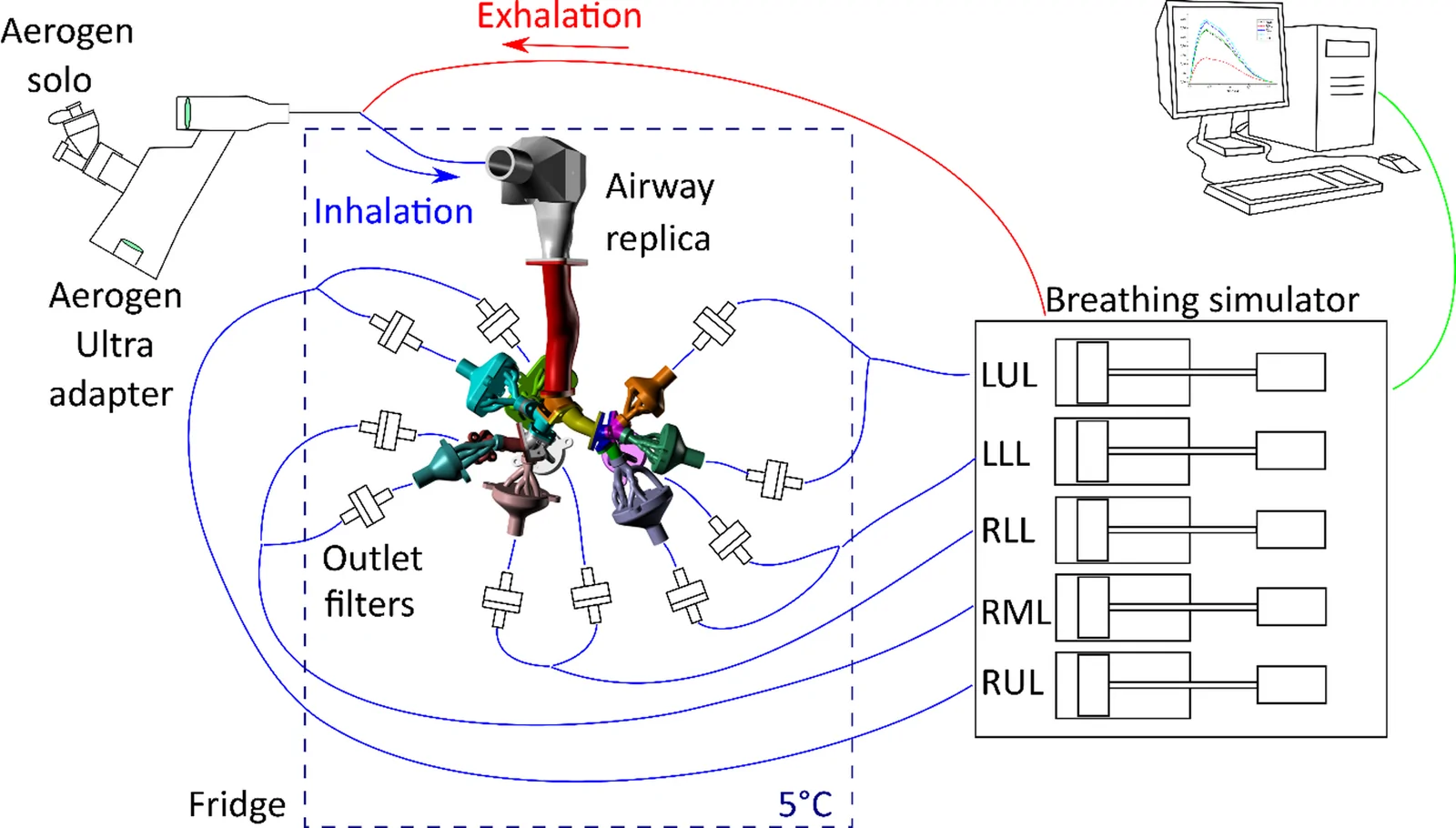
The scheme of the experimental setup used for deposition measurements.

The segmented airway replica was assembled and connected to the breathing simulator (the scheme is shown in Fig. 10, and a photograph of the actual experimental rig is provided in Supplementary Figure S1. All connections were sealed to prevent leaks. The replica was cooled to 5 °C to avoid droplet evaporation and to enable comparison with the ACI data. Nitrocellulose membrane filters (MF-Millipore™, pore size of 0.45 μm, diameter of 25 mm) were placed in filter holders and connected to each outlet of the airway replica to collect aerosol passing through the model’s branching levels. The model was connected to the inspiratory branches of the breathing simulator. Only inspiration was directed through the airway replica; expiration was vented in front of the Aerogen Ultra mouthpiece to prevent droplet accumulation and ensure realistic aerosol properties. The nebuliser was filled with 4 mL of FUR preparation (2 ampoules) and connected to the Aerogen Ultra adapter. The sampling duration consisted of 100 breathing cycles, during which the nebuliser generated aerosol continuously. After exposure, the airway model was disassembled, the deposited aerosol was extracted from the surfaces of each segment using methanol, and samples were analysed by HPLC to determine the mass fraction of FUR deposited in each segment. All deposition experiments were performed in triplicate, and the regional deposition fractions are reported as the mean ± SD.

###  Setup of the computational simulation

Numerical simulations using the CFD approach were performed in the commercial solver Simcenter Star-CCM+ v2210 Build 17.06.007. The simulation was carried out in three stages to replicate the conditions of the in vitro experiment as closely as possible. The first stage represented the inspiratory part of the respiratory cycle. During this phase, particles were injected into the domain at uniform time intervals, with diameters corresponding to the experimentally determined aerodynamic particle size distribution. Since the in vitro setup diverted expiratory flow away from the airway replica (as described in “Comparative lobar deposition in G4 and downwards (EXP vs. CFD vs. MPPD)”), the second stage of the simulation was defined as a period of zero airflow (simulating a breath hold within the model volume). During this stage, particle injection was disabled to mimic the absence of incoming aerosol during the expiratory phase. The duration of this stage corresponded to the expiratory time of the breathing cycle. Finally, because a non-negligible number of particles remained suspended in the domain after the second stage, a third stage consisting of a second inspiratory cycle was performed to allow the remaining particles to settle or exit the domain.

The geometry of the physical model used in the experiment was discretised using a computational mesh consisting of 10.93 million elements. The volume mesh was constructed using polyhedral cells, while the boundary layer was resolved using ten layers of prismatic elements. A traditional transient mesh independence test was not performed, as it is inherently problematic for LES. In LES, the grid explicitly defines the spatial filter; therefore, refining the mesh fundamentally alters the proportion of turbulence directly resolved versus modelled by the subgrid-scale model. As noted by Koullapis et al.^29^ in their computational assessment of upper airway flows (using the same airway geometry), this shift in subgrid model involvement can introduce variations—particularly in low-speed regions—that are related to subgrid turbulence modelling aspects rather than precluding numerical convergence.

Instead, a physics-based meshing strategy was adopted to ensure appropriate spatial resolution. Building on extensive previous validation studies utilising this exact geometry under various flow regimes^29,41^, a preliminary steady-state calculation was performed at the peak inspiratory flow rate. From this, the Taylor micro-scale was evaluated to determine the base cell size (0.8 mm) for the core mesh, ensuring that the large energy-containing eddies are directly resolved by the LES approach. The flow was assumed to be incompressible and isothermal. The calculation was solved as unsteady with an adaptive time step length to satisfy the Courant–Friedrichs–Lewy condition (CFL ≤ 1). Turbulence was modelled using LES with the Wall-Adapting Local Eddy-viscosity (WALE) subgrid-scale model with a time scale coefficient of 3.5^42^. The zero gauge pressure boundary condition was prescribed at the replica inlet. Outward-pointing velocities were set at the model outlets to a particular lobe to match the physiological flow distribution. A no-slip velocity condition was imposed on all airway walls. The conservation of mass and momentum was resolved using a segregated flow solver.

The particle deposition was solved by the multiphase Euler-Lagrangian approach, which solves the equation of motion for a spherical particle moving within the Eulerian continuum. The particle sizes were sampled based on the cumulative distribution function measured by ACI (as described in “MPPD modelling predictions”). The interaction between the continuous and dispersed phases was modelled using one-way coupling. The forces acting on the particles included gravity, the drag force calculated using the Schiller-Naumann correlation^43^, and the pressure gradient force. The particles were injected into the domain via 100 injection points uniformly distributed across the inlet of the mouthpiece adapter. During the inspiratory phase, 1.2 million particles were injected, and their transport and deposition were subsequently tracked through the remaining simulation phases.

## Supplementary Information

Below is the link to the electronic supplementary material.

Supplementary Material 1

## Data Availability

The datasets generated and/or analysed during the current study are available in the Zenodo repository, https://doi.org/10.5281/zenodo.18508754.
