# Supplementary material for: Aerodynamic barriers to inhaled furosemide delivery during dyspnoea revealed by experimental and computational modelling
Source: Sci Rep. 2026 Jun 8;16:26080. doi: 10.1038/s41598-026-55439-3 (PMC13490375; doi:10.1038/s41598-026-55439-3)
Supplement: Supplementary file 1 [file 41598_2026_55439_MOESM1_ESM.docx]

**Supplementary Information**

**to the**

**Aerodynamic barriers to inhaled furosemide delivery during dyspnoea revealed by experimental and computational modelling**

**by**

Frantisek Lizal, Miloslav Belka, Zdenka Sklubalova, Ondrej Misik, Jakub Elcner, Michal Svarc, Ludmila Matysova, Vladimir Koblizek

*Supplementary Figure S1: A photograph of the experimental setup. The airway replica with a connected nebuliser in the front, the breathing simulator in the background.*
